# An Investigation of Cognitive Performance and Electroencephalographic Correlations in a Healthy Elderly Sample

**DOI:** 10.1002/alz70857_104680

**Published:** 2025-12-25

**Authors:** Iany Tâmilla Pereira Batista, Keviny Magalhães Queiroz, Gustavo Coelho Peixoto, Carlos Eduardo de Souza Menezes, Arnaldo Aires Peixoto Junior, Edgar Marçal

**Affiliations:** ^1^ Federal University of Ceará, Fortaleza, Ceará, Brazil; ^2^ Christus University Center, Fortaleza, Ceará, Brazil

## Abstract

**Background:**

With the growth of the elderly population and the increasing incidence of diseases related to cognitive decline, there is a growing demand for effective cognitive screening methods. This study explores the application of a memory and learning test integrated with electroencephalographic (EEG) monitoring in healthy elderly individuals. The research aimed to investigate the correlation between cognitive performance and EEG changes during the test, providing a more comprehensive neurophysiological view of cognitive functions.

**Method:**

The study was carried out using the Rey Auditory Verbal Learning Test (RAVLT), designed to assess episodic declarative memory through auditory verbal learning. EEG was used to measure brain wave activity during the test, with Delta, Theta, Alpha, Beta and Gamma waves being recorded for later analysis. The experimental phase involved 18 healthy elderly participants.

**Result:**

Analysis of the EEG data revealed significant correlations between brainwave activity and cognitive performance. Specifically, higher levels of alpha activity were negatively correlated with immediate recall (rho = ‐0.529, *p* =  0.024) and delayed recall (rho = ‐0.593, *p* =  0.009), while higher levels of gamma activity were positively correlated with better performance in immediate recall (rho = 0.572, *p* =  0.013) and delayed recall (rho = 0.476, *p* =  0.046). Beta wave activity was negatively correlated with learning across trials during testing (rho = ‐0.536, *p* =  0.022).

**Conclusion:**

Cognitive testing combined with EEG monitoring shows promising potential for assessing cognitive function in the elderly. EEG integration allows deeper insights into the neurophysiological processes underlying cognitive performance. However, more studies are needed to validate its clinical applicability.

